# Supplementary material for: Evaluation of Serum Neurofilament Light Chain and Glial Fibrillary Acidic Protein as Screening and Monitoring Biomarkers for Brain Metastases
Source: Cancers (Basel). 2021 May 6;13(9):2227. doi: 10.3390/cancers13092227 (PMC8125258; doi:10.3390/cancers13092227)
Supplement: Supplementary file 1 [file cancers-13-02227-s001.zip › cancers-1184146-supplementary.pdf]

cancers

MDPI

Supplementary Materials

Evaluation of Serum Neurofilament Light Chain and Glial Fibrillary Acidic Protein as Screening and Monitoring Biomarkers for Brain Metastases

Su-Hyun Kim, Ho-Shin Gwak, Youngjoo Lee, Na-Young Park, Mira Han, Yeseul Kim, So-Yeon Kim and Ho Jin Kim

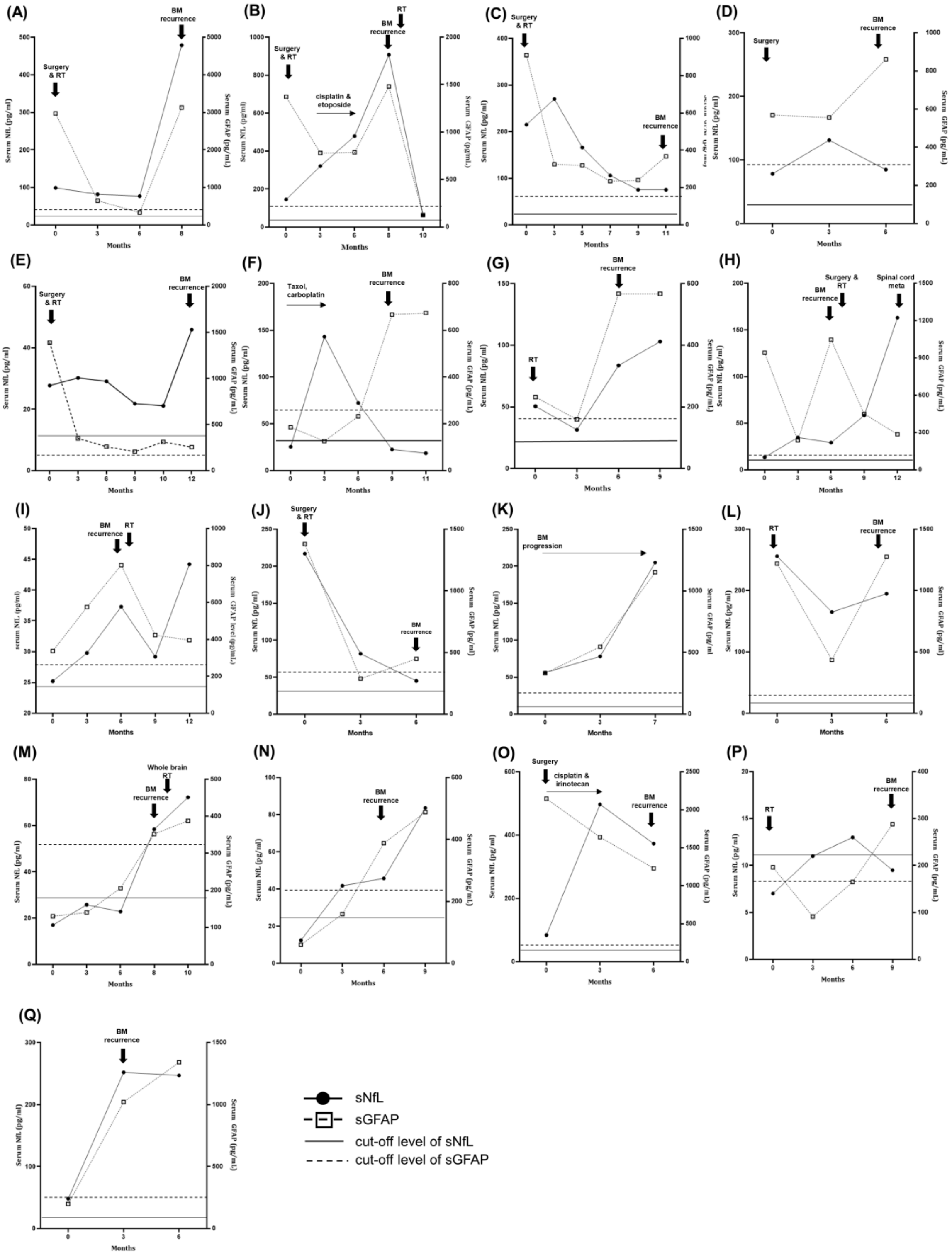

Figure S1. Longitudinal follow-up results of sNfL and sGFAP levels after BM diagnosis in 17 patients with BM progression. sNfL, serum neuro-filament light chain; sGFAP, serum glial fibrillary acidic protein; BM, brain metastasis.
